# Single‐cell RNA sequencing analysis of human kidney reveals the presence of ACE2 receptor: A potential pathway of COVID‐19 infection

**DOI:** 10.1002/mgg3.1442

**Published:** 2020-08-03

**Authors:** Qiyu He, Tsz N. Mok, Liang Yun, Chengbo He, Jieruo Li, Jinghua Pan

**Affiliations:** ^1^ First Affiliated Hospital of Jinan University Guangzhou Guangdong China; ^2^ Pediatric Cardiac Surgery Center National Center for Cardiovascular Disease and Fuwai Hospital Chinese Academy of Medical Sciences & Peking Union Medical College Beijing China; ^3^ First Medical College of Southern Medical University Guangzhou Guangdong China; ^4^ Heyu Health Technology Co Ltd. Guangzhou Guangdong China

**Keywords:** ACE2, COVID‐19, kidney, SARS‐Cov‐2, scRNA‐seq

## Abstract

**Background:**

A novel coronavirus called SARS‐Cov‐2, which shared 82% similarity of genome sequence with SARS‐CoV, was found in Wuhan in late December of 2019, causing an epidemic outbreak of novel coronavirus‐induced pneumonia with dramatically increasing number of cases. Several organs are vulnerable to COVID‐19 infection. Acute kidney injury (AKI) was reported in parts of case‐studies reporting characteristics of COVID‐19 patients. This study aimed at analyzing the potential route of SARS‐Cov‐2 entry and mechanism at cellular level.

**Method:**

Single‐cell RNA sequencing (scRNA‐seq) technology was used to obtain evidence of potential route and *ACE2* expressing cell in renal system for underlying pathogenesis of kidney injury caused by COVID‐19. The whole process was performed under R with Seurat packages. Canonical marker genes were used to annotate different types of cells.

**Results:**

Ten different clusters were identified and *ACE2* was mainly expressed in proximal tubule and glomerular parietal epithelial cells. From Gene Ontology (GO) & KEGG enrichment analysis, imbalance of *ACE2* expression, renin‐angiotensin system (RAS) activation, and neutrophil‐related processes were the main issue of COVID‐19 leading kidney injury.

**Conclusion:**

Our study provided the cellular evidence that SARS‐Cov‐2 invaded human kidney tissue via proximal convoluted tubule, proximal tubule, proximal straight tubule cells, and glomerular parietal cells by means of *ACE2*‐related pathway and used their cellular protease *TMPRSS2* for priming.

## Graphical abstract

Several organs are vulnerable to potential infection of COVID‐19 except lung, such as liver, digestive system, reproductive system and urinary system, and SARS‐CoV‐2 was separated from patients’ urine. Consequently, we hypothesized that COVID‐19 might invade human kidney causing renal injury, and this is the first study investigating the potential route of COVID‐19 leading kidney injury.

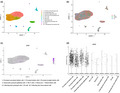

## 1 INTRODUCTION

In late December of 2019, a novel coronavirus was found in Wuhan (Chen et al., 2020), causing an epidemic outbreak of novel coronavirus‐induced pneumonia with dramatically increasing number of cases, 76,396 confirmed and 2,348 fatalities in China till 22nd February (She et al., 2020). It has been reported that there was 82% similarity of genome sequence between the SARS‐CoV and the novel coronavirus, which was named after SARS‐Cov‐2 by WHO (Chan et al., 2020; Van de Werf et al., 2012). This theory might indicate that SARS‐Cov‐2 infected human being via the same pathway as SARS‐CoV, *ACE2* (OMIM # 300335), and using cellular protease *TMPRSS2* (OMIM #602060) for priming (Hoffmann et al., 2020).

Apart from acute respiratory distress syndrome (ARDS) due to lung infection, other organs were revealed the potential risk of different human organs vulnerable to SARS‐Cov‐2 infection, such as lung, heart, digestive tract, and male reproductive system (Chai et al., 2020; Wang & Xu, 2020; Zhang et al., 2020; Zou et al., 2020). From a recent 138 hospitalized patients’ study, five acute kidney injury (AKI) (5/138, 3.6%) cases were reported, which might be caused by entry of SARS‐Cov‐2 through *ACE2* receptor resulting in kidney injury (Wang et al., 2020). Although previous studies (Mizuiri & Ohashi, 2015) had reported *ACE2* is expressed mainly in proximal tubules and glomeruli with the function of synthesis of inactive angiotensin 1–9 (Ang 1–9) from Angiotensin I (Ang I) and catabolism of Ang II to produce angiotensin 1–7 (And 1–7), which reduces vasoconstriction, water retention, salt intake, cell proliferation, reactive oxygen stress, and renoprotective effect. However, as the functional complexity of these structures appears to be associated with different cell types, the expression level, and function of *ACE2* in different cell types of human kidney is still unclear.

According to the study reporting kidney injury cases, direct effect of virus was suspected (Wang et al., 2020), and Academician Nanshan, Zhong, leader of high‐level steering team dealing with outbreak of COVID‐19 in China, declared that virus of COVID‐19, SARS‐Cov‐2, was separated from patients’ urine sample (Le, Knoedler, & Roberge, 2020). However, the potential route of SARS‐Cov‐2 entry and mechanism of kidney injury base on cellular level is unclear. Consequently, we hypothesize that SARS‐Cov‐2 may enter kidney by ACE2‐related pathway leading kidney injury. In this study, based on public databases, single‐cell RNA sequencing (scRNA‐seq) technology was used to obtain evidences of potential route of SARS‐Cov‐2 entry and underlying pathogenesis of kidney injury in COVID‐19 patients.

## 2 MATERIALS AND METHODS

### 2.1 Ethical compliance

This study does not include any participant or animal subjects so that the ethical compliance is not applicable.

### 2.2 Data sources

Gene expression matrix of normal human kidney were obtained from Gene Expression Omnibus (https://www.ncbi.nlm.nih.gov/geo/). scRNA‐seq raw data were obtained from Liao et al. (2020) (GSE131685), containing 23,366 high‐quality cells from three normal human kidney samples.

### 2.3 scRNA‐seq data processing and quality control

Whole process was performed under R (version 3.6.2) and the raw data of gene expression matrix was converted into Seurat object via the Seurat package of R (version 3.1.3). Average was acquired in the situation of duplicated gene expressions and low‐quality cells which had either expressed genes less than 200 or higher than 2500, or mitochondrial gene expression exceeded 30% were excluded for following analysis. Then, we visualized the relationships between the percentage of mitochondrial genes and mRNA reads, and between the number of mRNAs and the reads of mRNA. After that, remaining gene expression matrices were normalized and top 2,000 variable genes were selected for downstream analysis.

### 2.4 Principal component analysis (PCA) and dimensional reduction

Seurat *CellCycleScoring* function was administered to diminish the error in cell clustering since different phases of cell may interfere the following procedure and cell clustering results. All data were scaled to weight for downstream analysis and *RunPCA* function was used to determine PCs. By the visualization of JackStraw plot and Elbow plot, top 20 PCs were selected, and *RunHarmony* was adopted to eliminating batch effect as much as possible. *RunUMAP* as well as *FindNeighbors* function with harmony reduction was chosen and *FindClusters* with resolution of 0.25 was performed to identify different clusters.

### 2.5 Annotation of clusters and identification of ACE2 expression

Marker genes were found via *FindAllMarkers* function with threshold of 0.25 and annotation was performed based on the canonical marker genes provided by previous studies (Chabardès‐Garonne et al., 2003; Chen, Cheung, Shi, Zhou, & Lu, 2018; Habuka et al., 2014; Lee, Chou, & Knepper, 2015; Nakagawa et al., 2002; Park et al., 2018; Shankland, Smeets, Pippin, & Moeller, 2014; Young et al., 2018). After cell type identification, violin plot as well as feature plot were visualized to identify the *ACE2* expression in each cluster. Also, human protein atlas of *ACE2* was accessed in order to acquire more evidence ascertaining the expression level of *ACE2* in kidney cells (https://www.proteinatlas.org/). In addition, co‐expression analysis was performed with normalized cluster matrices data. Pearson's correlation test was administered between each gene in single‐cell transcriptome and *ACE2*. Significantly top 200 *ACE2* co‐expressed genes (*p* value <0.05) were collected for following downstream Gene Ontology (GO) as well as Kyoto Encyclopedia of Genes and Genomes (KEGG) enrichment analysis (*p* value <0.05), with the corresponding databases of Carlson M (2019). *org*.*Hs*.*eg*.*db*, and *STRINGI*, respectively.

## 3 RESULTS

### 3.1 Identification of cell types

**Figure 1:**
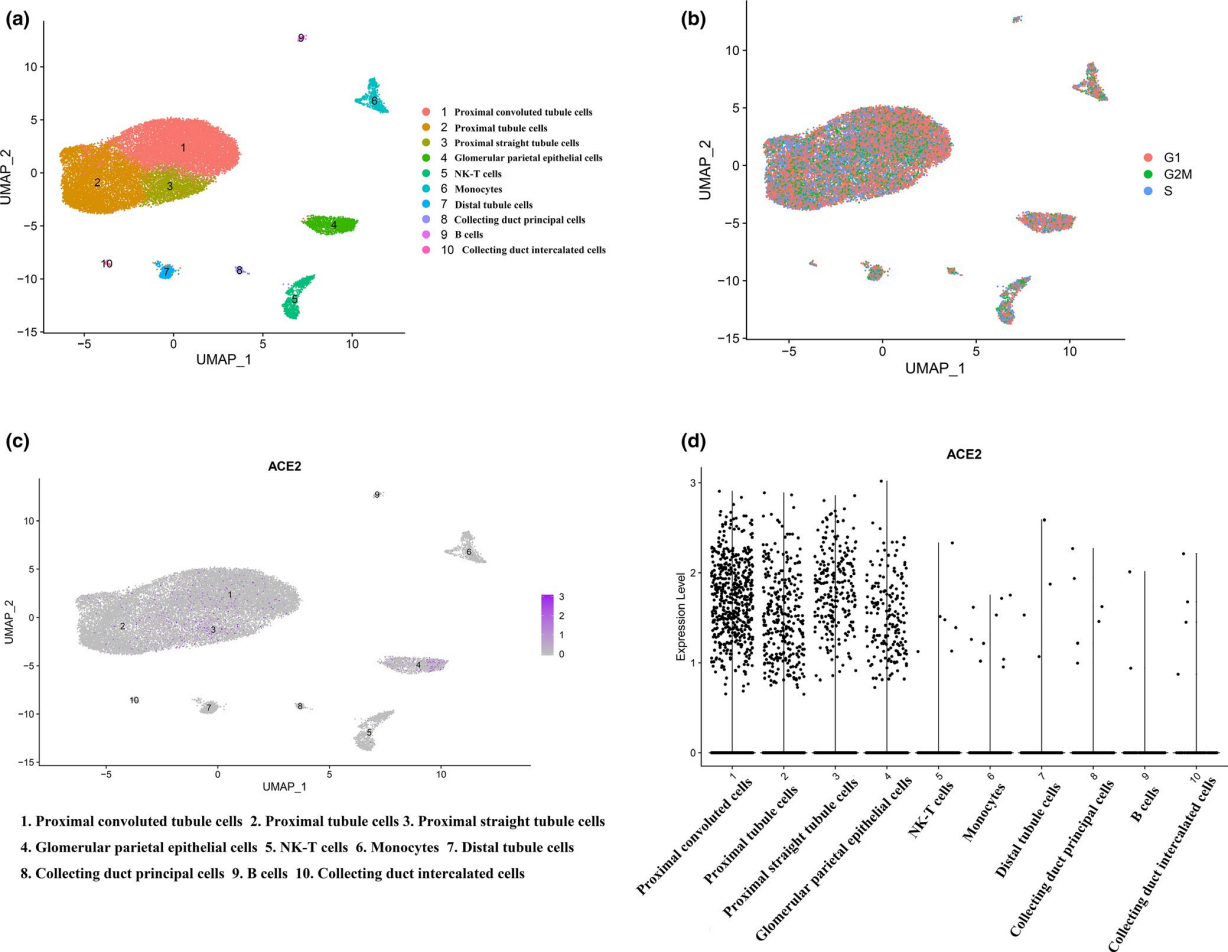
Results from scRNA‐seq analysis. (a) Uniform manifold approximation and projection (UMAP) plot of samples revealing the different clusters of renal cells. (b) UMAP plot indicating different cluster with demonstration of different cell cycles. (c) Expression of ACE2 in different clusters of cells. (d) Violin plot showing the ACE2 expression in different clusters of cells. scRNA, single‐cell RNA sequencing

After quality control and data normalization among three human kidney samples (baseline characteristics of three samples are documented in Table S1) (Liao et al., 2020), a total of 17,528 RNA features and 23,367 cells were retained for downstream analysis (detail of initial sequencing data is shown in Figure S1). Uniform Manifold Approximation and Projection (UMAP) was used for dimensional reduction for 23,367 high‐quality cells and 10 different clusters were obtained eventually (Figure 1a). In order to reduce interference of cell clustering due to diverse phase in a cell cycle, *CellCycleScoring* function was used and visualization of no bias induced by cell cycle genes was observed (Figure 1b). According to differential expressed genes in each cluster (Table S2) and canonical marker genes provided by Liao et al. (Table S3), 10 clusters were annotated as proximal convoluted tubule cells, proximal tubule cells, proximal straight tubule cells, glomerular parietal epithelial cells, NK‐T cells, monocytes, distal tubule cells, collecting duct principal cells, B cells, and collecting duct intercalated cells, respectively.

### 3.2 Specific expression of ACE2

**Figure 2:**
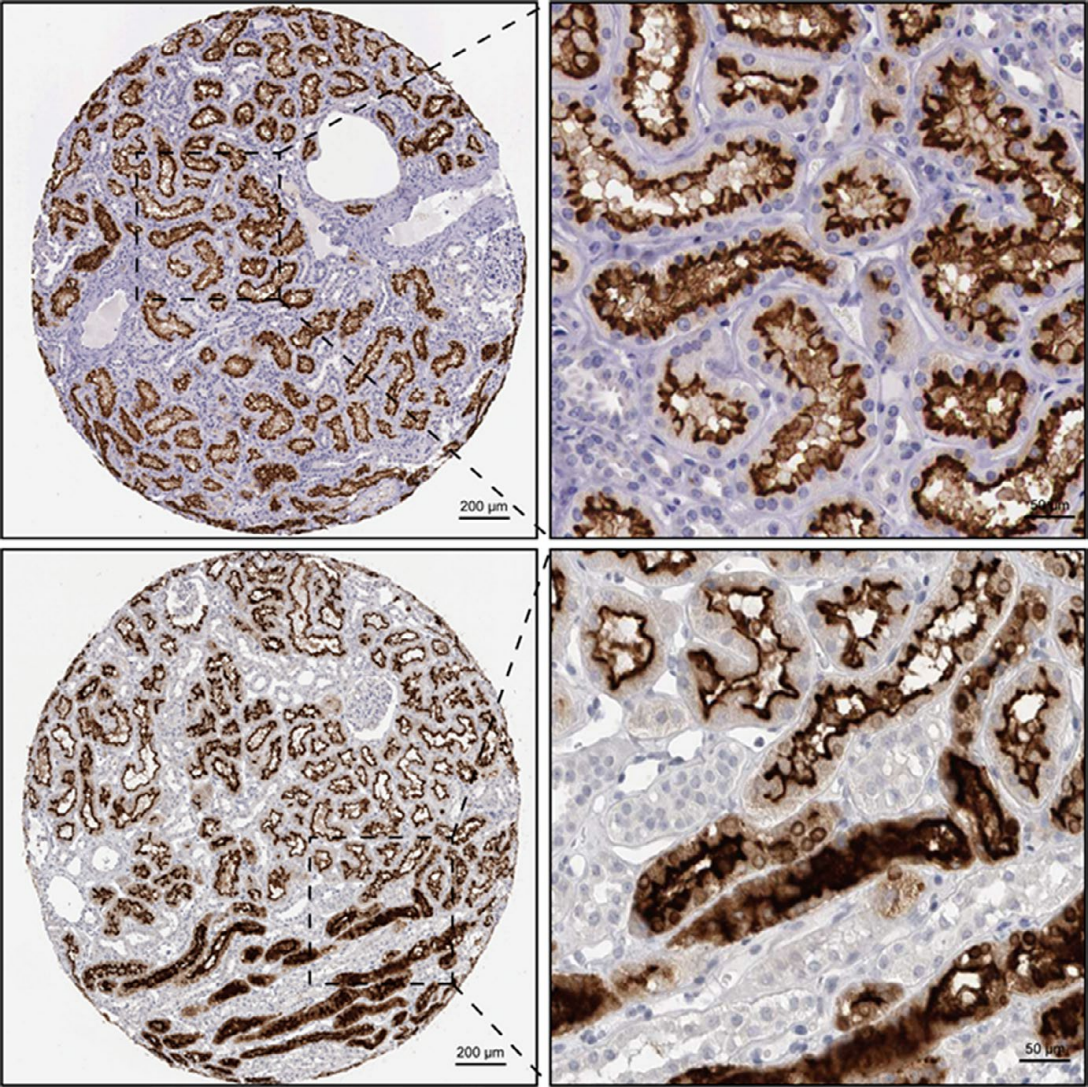
Human protein atlas showing the expression level of ACE2. (Shade of brown indicates the expression level: darker indicates high‐level expression and lighter indicates low level expression)

*ACE2* expression level in each cluster was identified by violin plot as well as scatter plot with reduction of UMAP, revealing that *ACE2* was predominantly expressed in proximal convoluted tubule cells, proximal tubule cells, proximal straight tubule cells, and glomerular parietal epithelial cells, respectively (Figure 1c,d). The human protein atlas of *ACE2* in kidney cells indicated that a majority of *ACE2* expression located in proximal convoluted and straight cells (Figure 2). As we discussed in introduction, SARS‐Cov‐2 entered human body via *ACE2*‐related pathway using cellular protease TMPRSS2 for priming (Hoffmann et al., 2020). We additionally determined the expressing level of *TMPRSS2* in each cell cluster and, it was mainly expressed in cluster one to four and seven to eight (Figure S2), which are proximal convoluted tubule, proximal tubule, proximal straight tubule cells and glomerular parietal epithelial cells, distal tubule cells, and collecting duct principal cells, respectively. Therefore, with the intersection of ACE2 expressing level in each cell clusters and corroborative evidence from human protein atlas of ACE2, it furtherly provided substantial evidence to ascertain the potential entry pathway of SARS‐Cov‐2 by invading proximal tubule cells as well as glomerular parietal epithelial cells and, it gave a latent theory of SARS‐Cov‐2 replicating within these cells by using cellular protease TMPRSS2 for priming.

### 3.3 GO & KEGG analysis of ACE2 co‐expression genes

Pearson's correlation test was administered between *ACE2* and cluster one to four to obtain significantly positive or negative co‐expressed genes (*p* value <0.05). Top 200 positive and negative co‐expressed genes (top 50 is listed in Table 1) were selected to downstream GO and KEGG analysis (Complete *ACE2* co‐expressed genes are documented in Table S4).

**Table 1:** Positive and negative co‐expression gene of ACE2

Cluster 1 (+)	Cluster (−)	Cluster 2 (+)	Cluster 2 (−)	Cluster 3 (+)	Cluster 3 (−)	Cluster 4 (+)	Cluster 4(−)
TMBIM6	RPS29	MTRNR2L1	RPL21	FAM118A	GSTP1	GPRIN2	EIF1
CLDN2	RPL21	PSAP	MT1H	THSD4	MT1E	SAPCD1‐AS1	HLA‐B
DPEP1	RPS27	MTRNR2L10	RPL41	SLC22A7	TMSB10	GAB1	B2M
NAT8	RPL41	MTRNR2L8	RPS27	GLYATL1	ACTG1	TTLL7	RPL18
HSP90B1	RPL12	TMBIM6	RPS29	DCXR	KIFC3	STS	ARHGDIB
SLC27A2	RPS26	EPCAM	RPL12	ABHD14A	NEAT1	MFSD2A	RPL4
ITM2B	RPS4Y1	MTRNR2L6	RPL39	BTG2	ITGAV	EPDR1	TMSB4X
MTRNR2L1	FXYD2	SLC3A1	MT1G	NRSN2‐AS1	CHMP4A	FARP2	RPS3
SPINT2	MT1G	GAL3ST1	RPL26	ASPHD2	WFDC2	C9orf116	RPL41
APOM	RPL26	CDH16	MT1F	NAT8	MYL6	LINC00526	HLA‐A
CLTRN	MT1H	MTRNR2L11	ATP5F1E	MYH8	PTPN1	SPTBN4	EVL
SLC22A6	UQCRQ	SLC27A2	POLR2L	CYP4A11	MBD4	SBSPON	TRBC2
CYBA	S100A1	PTH1R	MT2A	AZGP1	POLR2J3	ENPP7	
TMED10	UQCR11	GMFG	COX7C	MSRB1	SOX4	SH3BGRL2	
GADD45A	RARRES3	APOM	RPS4Y1	PCK1	GPAT4	MGAM	
STARD9	MIF	ATP1A1	IFITM3	MTRNR2L1	CGNL1	ST6GALNAC3	
TPT1	FABP3	PLG	FXYD2	DEPDC1	LRRFIP1	NFASC	
FOLR1	S100A11	ERRFI1	UBA52	F11‐AS1	IFITM3	KLHL29	
RTN4	NDUFA13	SLC22A6	RPL31	RBP4	PPP1R14B	EMILIN1	
SLC3A1	DAB2	BHMT	MT1E	GSTA2	PSMD12	TANC1	
SLC13A3	SVIP	CYBA	RPS26	GSTA5	TAX1BP3	PLA2R1	
CTBP1‐AS	RPL39	SLC22A11	NDUFB1	SMIM24	ABHD11	PARD3B	
MTRNR2L8	ATP5ME	PCK1	UQCR11	CARMN	AC124319.1	COL4A3	
CUBN	RPL31	AZGP1	MIR4458HG	ACVR1	RABIF	MYRIP	
PSAP	PSME1	PCP2	TMSB10	AKR7A3	CITED4	TNNC1	
SERPINI1	RPS8	CYP4F2	RPS2	MIOX	EPS15	RBP1	
AQP1	SYF2	GRK4	RPS15A	RTN4	MIEN1	PLOD2	
FAM151A	NDUFA1	ADAMTSL5	SKP1	MTRNR2L10	THBS1	APOD	
GGH	PCP4	DDC	PPDPF	GPT	RDH11	BASP1	
ATP1A1	POLR2L	FBXL6	PDCD5	GADD45A	ARHGAP35	SPOCK1	
SLC6A19	RPL36A	CES2	RPLP2	ARHGAP24	GOLGA2	LRP11	
SLC22A2	RPL37A	IWS1	APEX1	RGN	WDR83	MAGI2	
C18orf54	ATP5MF	ATP6V0B	CCDC58	ARFGEF3	EXOSC7	BGN	
SMIM24	COX7C	ADIRF	MACROD1	ALDH2	MBNL1	WT1	
TMEM59	NDUFA3	MEPCE	SULT1A1	MPC1	BNC2	CDC42EP2	
NACA2	OST4	PDIA3	NME1	ATP1B1	NKAIN4	ZNF385A	
CDHR2	NIPSNAP2	ASAH1	IL32	SEC14L2	RNPC3	DCN	
FN3K	MICOS10	MTRNR2L12	RPS19	EHMT2‐AS1	C3	RHBDF1	
GOLGA8A	TOMM5	NAT8	MTLN	FMO5	EIF4A3	VASN	
PLXNA3	HSPA1B	AGT	RPL19	GGH	YPEL3	ZNF423	
AUP1	DCDC2	IRX5	RPS28	SLC39A5	SNX6	TPPP3	
SLC3A2	OXT	ZNF786	RPL23A	TMEM176B	POLR2L	CA10	
APOE	RPL17	PTN	PSMA2	CERCAM	ZSCAN16‐AS1	NPHS1	
SARAF	WFDC2	ABHD14A	S100A1	RNF165	MT‐ND4	CLIP3	
SPP1	ATP5MPL	SPINT2	CAPG	GC	ANXA4	CXADR	
AZGP1	DYNC1I2	SEC23IP	AC026462.3	PLCH2	VPS35	UACA	
CD63	MT1E	FN3K	EIF3J	ADIRF	BNIP2	CDK2AP1	
AMN	AL359555.4	DHRS7B	PCP4	TEX51	RAB6A	PPP1R14C	
MAGEE1	S100A10	ZBTB8A	NOB1	EPHA1‐AS1	MIR29B2CHG	HAAO	
MTRNR2L10	RGS14	IMPDH1	ATP5ME	EXTL3‐AS1	PAFAH1B3	CDC42BPB	

Note

Top 50 genes of each (Total co‐expression genes could be checked in supplementary file).

“+” indicates positive co‐expressed genes of ACE2.

“−” indicates negative co‐expressed genes of ACE2.

**Figure 3:**
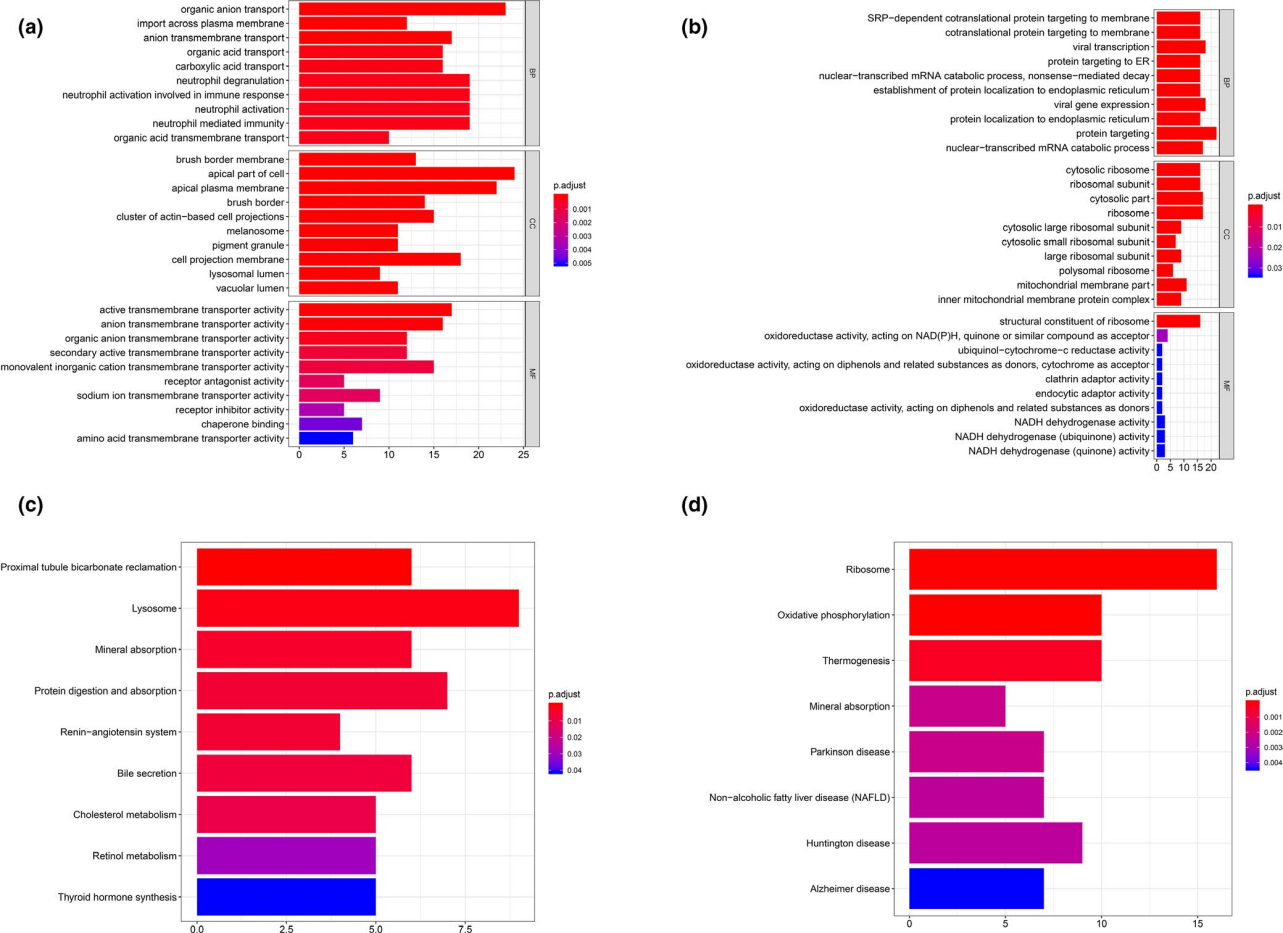
GO & KEGG analysis of cluster 1. (a) GO analysis of positive top 200 co‐expression genes of ACE2 in cluster 1. (b) GO analysis of negative co‐expression genes of ACE2 in cluster 1. (c) KEGG analysis of positive top 200 co‐expression genes of ACE2 in cluster 1. (d) KEGG analysis of negative top 200 co‐expression genes of ACE2 in cluster 1. GO, Gene Ontology

Regarding to cluster 1, proximal convoluted tubule cells, material transportation processes (*e*.*g*.* CLN3 [OMIM #607042]*,* SLC7A2 [OMIM #601872]*,* SLC2A6 [OMIM #606813]*,* NPC2 [OMIM #601015]*), transportation‐related activity, and cellular components (*e*.*g*.* PCYOX1 [OMIM #610995]*,* TIMM23B [OMIM #605034]*,* SLC2A6*,* SLC9A3 [OMIM #182307]*), and neutrophil‐mediated immunity (*e*.*g*.* METTL7A [OMIM #618338]*,* DYNLL1 [OMIM #601562]*,* S100P [OMIM #600614]*,* CST3 [OMIM #604312]*) were enriched in positive co‐expressed GO analysis (Figure 3a) and, renin‐angiotensin system (RAS) was revealed significance in KEGG pathway analysis (Figure 3c). For negative co‐expressed genes, viral transcription and expression, mRNA catabolic processes (*e*.*g*.* RPS29 [OMIM #603633]*,* RPL21 [OMIM #603636]*,* RPS27 [OMIM #603702]*,* RPL41 [OMIM #613315]*) were enriched in GO analysis (Figure 3b), and ribosome as well as oxidative phosphorylation pathway were enriched in KEGG analysis (Figure 3d).

**Figure 4:**
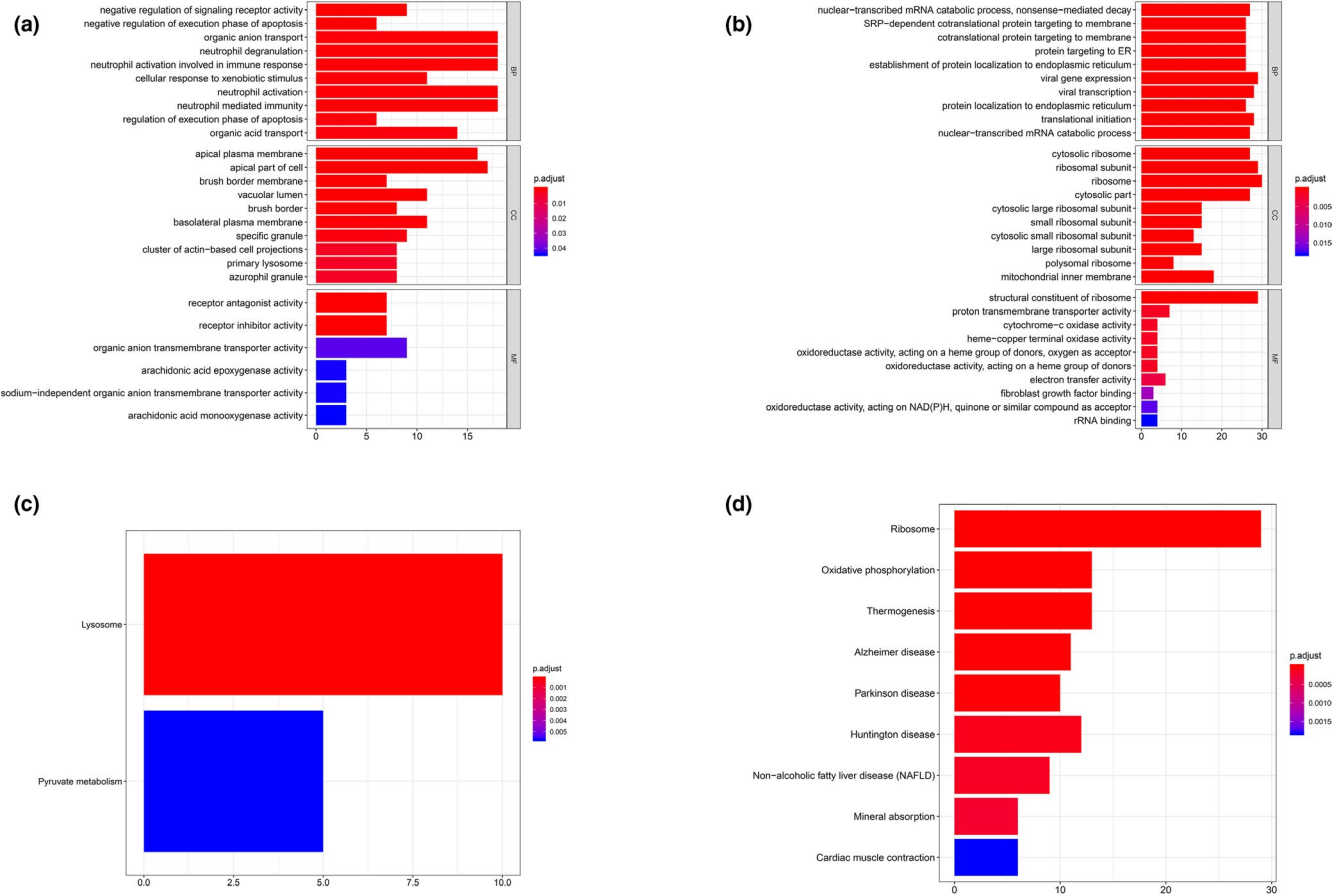
GO & KEGG analysis of cluster 2. (a) GO analysis of positive top 200 co‐expression genes of ACE2 in cluster 2. (b) GO analysis of negative co‐expression genes of ACE2 in cluster 2. (c) KEGG analysis of positive top 200 co‐expression genes of ACE2 in cluster 2. (d) KEGG analysis of negative top 200 co‐expression genes of ACE2 in cluster 2. GO, Gene Ontology

In cluster 2, proximal tubule cells, except material transportation enriched biological processes as cluster 1, neutrophil activation, immune‐related responses and degranulation (*e*.*g*.* MLEC [OMIM #613802]*,* CTSH [OMIM #116820]*,* PRCP [OMIM #176785]*,* UNC13D [OMIM #608897]*) were enriched for positive co‐expression GO terms (Figure 4a), and lysosome‐related pathway was shown in KEGG analysis (Figure 4c). Contrarily, in negative co‐expression enrichment, viral gene transcription, expression, and translational initiation were dominant in GO analysis (*e*.*g*.* RPL21*,* RPL41*,* RPS27*,* RPS29*) (Figure 4b), while in KEGG analysis, ribosome‐related pathway was enriched (Figure 4d).

**Figure 5:**
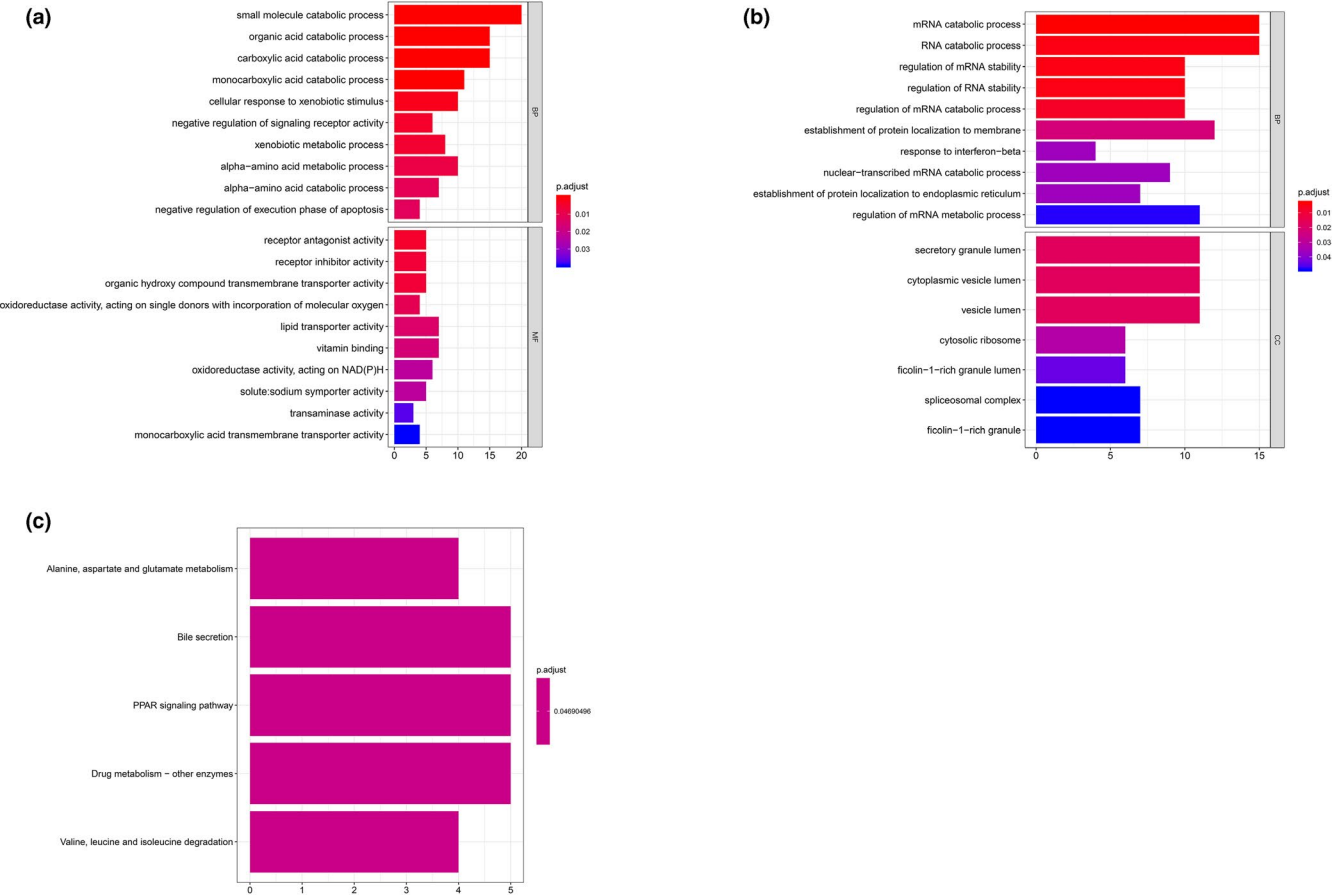
GO & KEGG analysis of cluster 3. (a) GO analysis of positive top 200 co‐expression genes of ACE2 in cluster 3. (b) GO analysis of negative co‐expression genes of ACE2 in cluster 3. (c) KEGG analysis of positive top 200 co‐expression genes of ACE2 in cluster 3. (d) KEGG analysis of negative top 200 co‐expression genes of ACE2 in cluster 3. GO, Gene Ontology

When it comes to proximal straight tubule cells of cluster 3, material catabolic and metabolic process (*e*.*g*.* AGXT2 [OMIM #612471]*,* ASRGL1 [OMIM #609212]*,* HPD [OMIM #609695]*,* HYAL1 [OMIM #607071]*) were predominantly enriched GO terms in top 200 positive co‐expressed genes (Figure 5a) with peroxisome proliferators‐activated receptors (PPAR) signaling pathway KEGG analysis shown in Figure 5c. Nevertheless, for negative co‐expressed genes, RNA‐related catabolic processes and regulation (*e*.*g*.* PSMD12 [OMIM #604450]*,* EXOSC7 [OMIM #606488]*,* EIF4A3 [OMIM #608546]*,* PSMD13 [OMIM #603481]*) were mainly enriched for GO terms (Figure 5b) while there was no significant KEGG pathway could be obtained.

**Figure 6:**
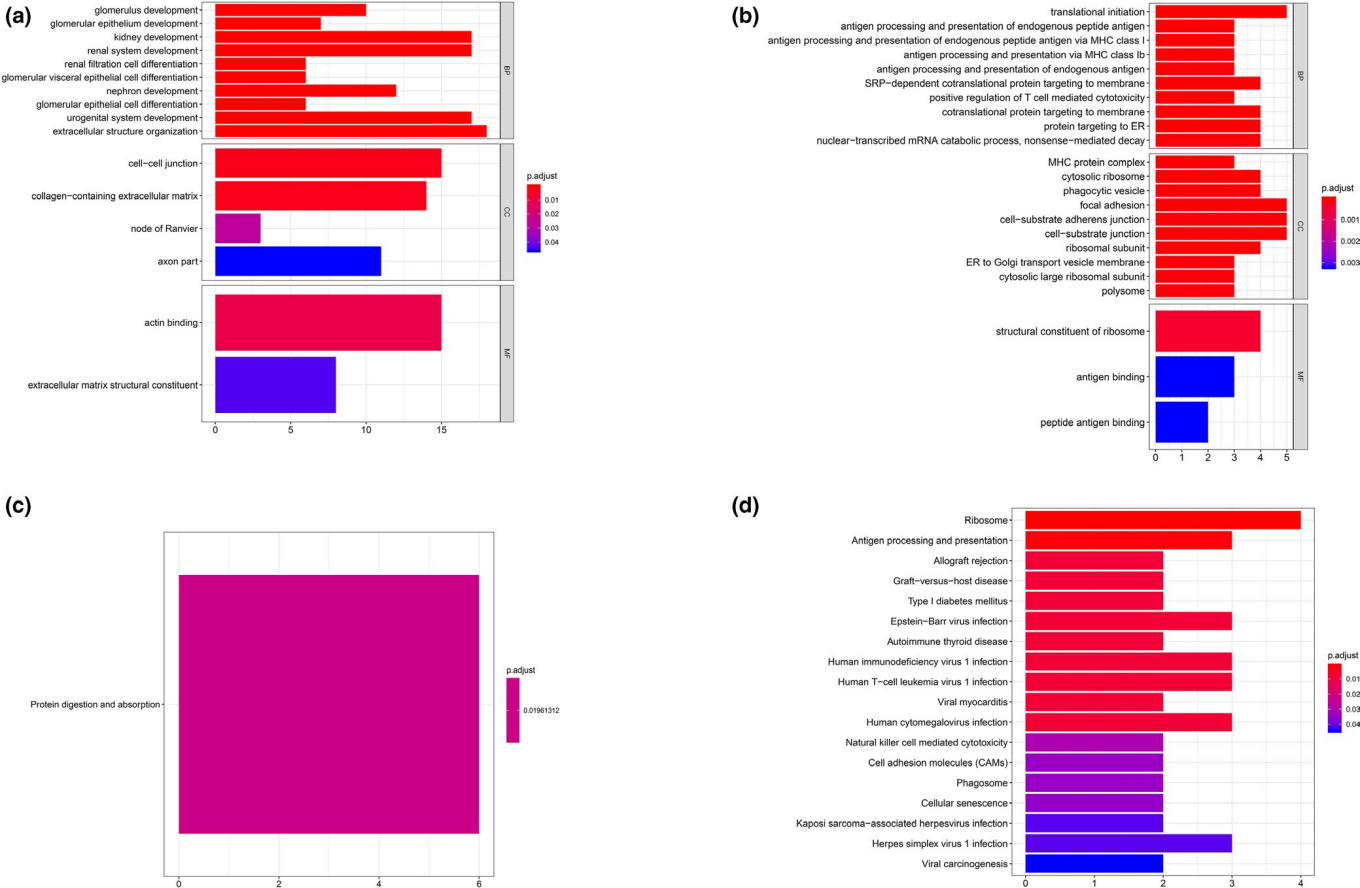
GO & KEGG analysis of cluster 4. (a) GO analysis of positive top 200 co‐expression genes of ACE2 in cluster 4. (b) GO analysis of negative co‐expression genes of ACE2 in cluster 4. (c) KEGG analysis of positive top 200 co‐expression genes of ACE2 in cluster 4. (d) KEGG analysis of negative top 200 co‐expression genes of ACE2 in cluster 4. GO, Gene Ontology

For glomerular parietal epithelial cells of cluster 4, renal cell differentiation, urogenital system development, and extracellular structure organization (*e*.*g*.* CDKN1C [OMIM #600856]*,* BMP7 [OMIM #112267]*,* MME [OMIM #120520]*,* LAMA5 [OMIM #601033]*)‐related GO terms were enriched among positive co‐expressed genes (Figure 6a) and, protein digestion as well as absorption pathway were enriched for KEGG‐related analysis (Figure 6c). Conversely, among negative co‐expressed genes GO analysis, translational‐related processes and antigen presentation processes (*e*.*g*.* EIF1 [OMIM #300186]*,* RPL18 [OMIM #604179]*,* HLA*‐*B [OMIM #142830]*,* B2 M [OMIM #109700]*,* HLA*‐*A [OMIM #142800]*) were enriched GO terms (Figure 6b), while ribosome and viral infection‐related pathways were enriched in KEGG analysis (Figure 6d).

## 4 DISCUSSION

According to previous study (Chiu, 2003; Yeung et al., 2016), two categories of coronavirus, SARS‐CoV and MERS‐CoV had been reported to damaged kidney through immunocompromise and induced apoptosis of renal cells. Although the exact evidence for kidney injury caused by COVID‐19 has not been estimated, several infected patients’ studies revealed the potential risk of kidney vulnerable to be damaged due to SARS‐Cov‐2 virus direct effect. Wang et al. (2020) reported five AKI cases in total of 138 patients, Huang et al. (2020) reported three AKI cases which were all in intensive condition, Guan et al. (2020) reported six AKI patients among total of 1,099 patients, and three patients with creatinine level greater than 133 μmol/L, indicating suspecting kidney injury, in total of 62 patients outside Wuhan were reported by Xu et al. (2020). Hypothesis of virus direct effect resulting in kidney damage was suspected within the abovementioned case‐studies and, more importantly, based on our study results of scRNA‐seq in human kidney, potential route for COVID‐19 leading kidney damage via *ACE2* was estimated.

*ACE2* participates in the synthesis and catabolism of Ang II that induces inflammation, cell growth, mitogenesis, apoptosis, and regulates the gene expression of bioactive substances, all of which might exacerbate to renal tissue injury. Moreover, ACE2 inhibition was reported to be associated with increasing albumin excretion and worsen glomerular injury (Soler, Wysocki, & Batlle, 2008), which was confirmed by an experimental study using exogenous human recombinant *ACE2* to slow the progression of chronic kidney disease by reducing in albumin excretion (Oudit et al., 2010). As we known, catabolism of Ang II to produce Ang 1–7 is the main function of *ACE2*, while renal damage or disorder may happen with increasing Ang II (Ruiz‐Ortega et al., 2006). Thus, the imbalance expression level of *ACE2* accelerates the renal damage.

Furthermore, RAS could be a potential pathway influencing on renal injury progression via mitogen‐active protein kinase (MAPK)‐mediated apoptosis, NF‐κB‐mediated inflammation, and redox imbalance promoting oxidative stress (Gnudi, Coward, & Long, 2016; Newsholme, Cruzat, Keane, Carlessi, & de Bittencourt, 2016; Sharma, Anders, & Gaikwad, 2019). A literature speculated that compromised kidney perfusion and altered intrarenal hemodynamics balance attributed to systemic and intrarenal RAS activation may be the critical pathogenic factors (Matejovic et al., 2016). From our GO & KEGG analysis, RAS was a significant pathway in *ACE2* co‐expressed gene enrichment, which might indicate one of the potential mechanisms of COVID‐19 damage to kidney. Interestingly, neutrophil‐mediated immunity was detected in almost positive co‐expressed genes analysis. SARS‐Cov‐2 invasion to kidney via *ACE2* may interfere immunity response, which was a underlying mechanism similar to SARS‐CoV damage to kidney by immunocompromise (Chiu, 2003). Glomerulus was also a potential target for SARS‐Cov‐2 according to our scRNA‐seq data, and the GO enrichment of glomerulus was associated with development, such as glomerulus development, nephron development, even though kidney development, which may be a pathogenic factor for the exacerbation of patient kidney's status.

To the best of our knowledge, no specific medication could be used to against COVID‐19 but symptomatic treatment would be the predominant strategy. Occurrence of AKI is the most frequent renal‐related complication in COVID‐19 patients, which increases the risk factor for mortality. From above discussion, dealing with *ACE2* expression imbalance and RAS activation would be the main issues. Recombinant *ACE2* was reported to be effective in slowing the progression of kidney disease by reducing albumin excretion and modulating RAS depressor arm (*AT2R*, *ACE2*, Ang 1–7) by *ACE2* activator or *AT2R* agonist might protect the kidney from renal injury (Oudit et al., 2010; Sharma, Malek, Mulay, & Gaikwad, 2019), which may provide potential clues for clinicians encountering COVID‐19 patients, especially complicated with kidney injury. However, the abovementioned mechanism of kidney injury in COVID‐19 infection should be verified in experiment under precise experimental design.

## 5 CONCLUSION

This study speculated that SARS‐Cov‐2 invaded proximal convoluted tubule, proximal tubule, proximal straight tubule cells, and glomerular parietal epithelial cells by means of *ACE2* receptor and damaging kidney tissue through *ACE2* imbalance and RAS activation, which offers substantial clues for clinical practice dealing with renal‐related complications caused by COVID‐19.

## CONFLICT OF INTEREST

All authors declared no conflicts of interest to disclose.

## AUTHOR CONTRIBUTIONS

The detailed contributions of each authors are listed as followed: Qiyu He: conceptualization, methodology, data analysis, manuscript writing. Tsz Ngai Mok: methodology, data analysis, manuscript writing. Liang Yun: investigation. Chengbo He: technical support of data processing. Jieruo Li: supervision, conceptualization, professional suggestion and revision. Jinghua Pan: supervision, conceptualization, professional suggestion and revision.

## Supporting information

Fig S1

Fig S2

Table S1

Table S2

Table S3

Table S4
