# Supplementary figures and images for: Single‐cell RNA sequencing analysis of human kidney reveals the presence of ACE2 receptor: A potential pathway of COVID‐19 infection
Source: Mol Genet Genomic Med. 2020 Aug 3;8(10):e1442. doi: 10.1002/mgg3.1442 (PMC7435545; doi:10.1002/mgg3.1442)

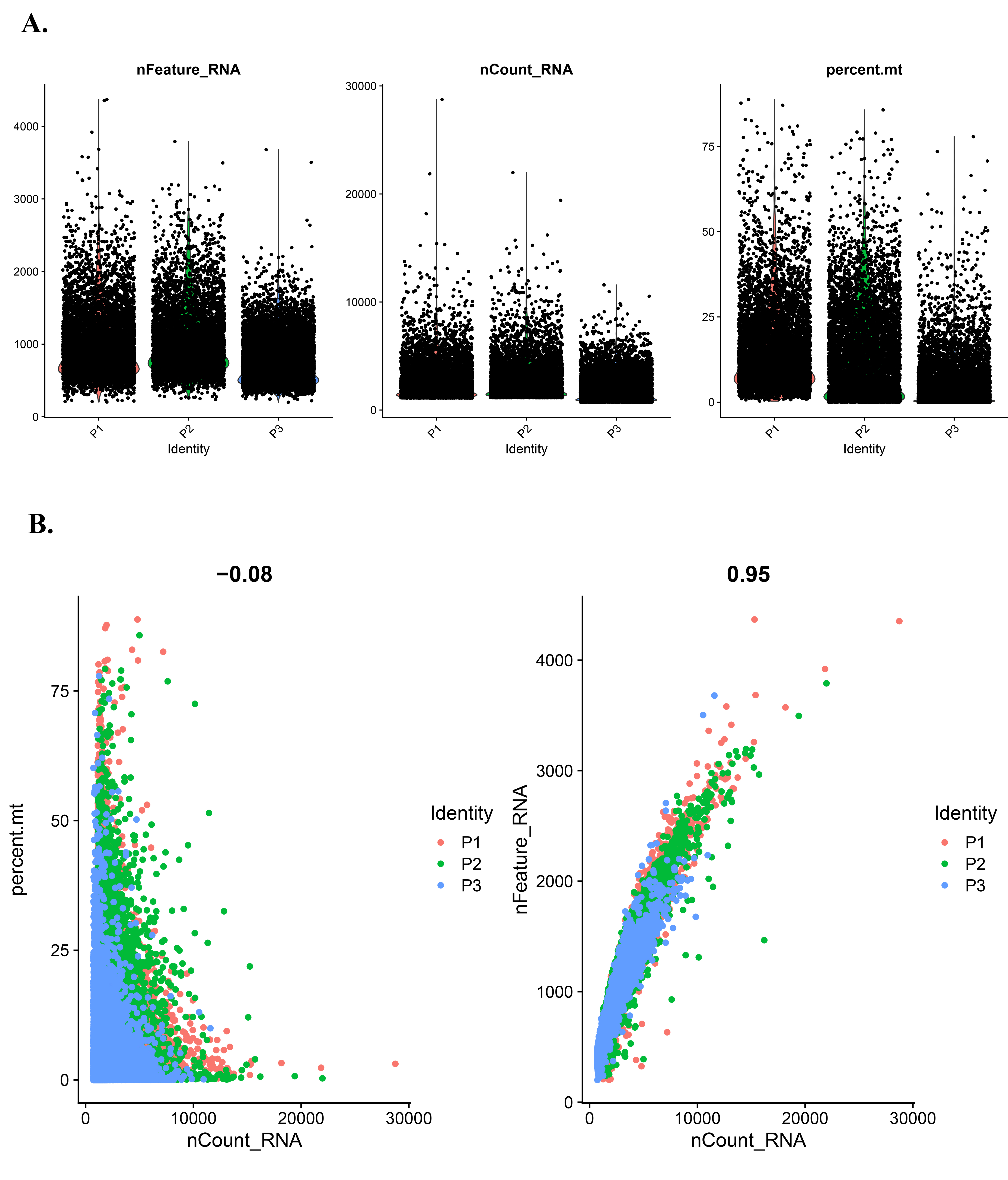

Supplement: Supplementary file 1 — Fig S1 [file MGG3-8-e1442-s001.jpg]

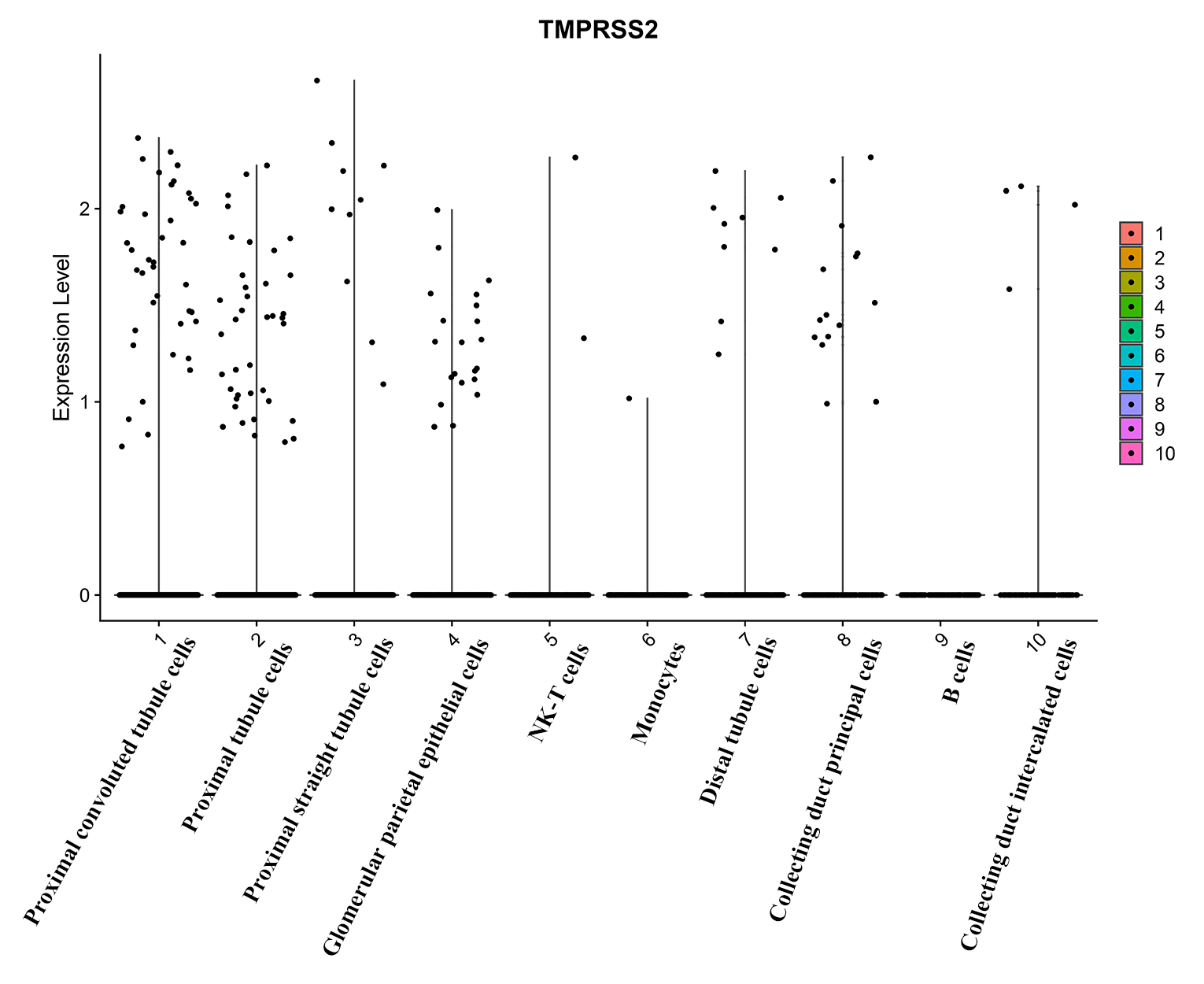

Supplement: Supplementary file 2 — Fig S2 [file MGG3-8-e1442-s002.jpg]
