# Supplementary material for: Single‐cell RNA sequencing analysis of human kidney reveals the presence of ACE2 receptor: A potential pathway of COVID‐19 infection
Source: Mol Genet Genomic Med. 2020 Aug 3;8(10):e1442. doi: 10.1002/mgg3.1442 (PMC7435545; doi:10.1002/mgg3.1442)
Supplement: Supplementary file 3 — Table S1 [file MGG3-8-e1442-s003.docx]

**Table S1. Baseline information of samples**

| **ID** | **Age (yr)/Sex** | **Histology** | **Smoker/Drinker** | **Height/Weight (cm/kg)** | **Type of sample** |
| --- | --- | --- | --- | --- | --- |
| Kidney 1 | 57/M | Papillary urothelial carcinoma of the renal pelvis | Yes/No | 170/74 | Normal kidney |
| Kidney 2 | 59/F | Clear renal cell carcinoma | No/No | 152/47 | Normal kidney |
| Kidney 3 | 65M | Clear renal cell carcinoma | Yes/No | 162/59 | Normal kidney |
