# Supplementary material for: Single‐cell RNA sequencing analysis of human kidney reveals the presence of ACE2 receptor: A potential pathway of COVID‐19 infection
Source: Mol Genet Genomic Med. 2020 Aug 3;8(10):e1442. doi: 10.1002/mgg3.1442 (PMC7435545; doi:10.1002/mgg3.1442)
Supplement: Supplementary file 5 — Table S3 [file MGG3-8-e1442-s005.docx]

**Table S2. Canonical marker genes of kidney cells**

| **Gene** | **Marker** |
| --- | --- |
| *SLC13A3* | Proximal tubule |
| *SLC34A1* | Proximal tubule |
| *GPX3* | Proximal tubule |
| *DCXR* | Proximal tubule |
| *SLC22A8* | Proximal convoluted tubule |
| *SLC22A7* | Proximal straight tubule |
| *KRT8* | Glomerular parietal epithelial cells |
| *KRT18* | Glomerular parietal epithelial cells |
| *CD24* | Glomerular parietal epithelial cells |
| *VCAM1* | Glomerular parietal epithelial cells |
| *LYZ* | Monocytes |
| *CD14* | Monocytes |
| *GNLY* | NK cells |
| *NKG7* | NK cells |
| *CD3D* | T cells |
| *CD3E* | T cells |
| *IL7R* | T cells |
| *CD79A* | B cells |
| *CD79B* | B cells |
| *UMOD* | Distal tubule |
| *DEFB1* | Distal tubule, Collecting duct |
| *CLDN8* | Collecting duct |
| *AQP2* | Collecting duct principal cells |
| *ATP6V1G3* | Collecting duct intercalated cells |
| *ATP6V0D2* | Collecting duct intercalated cells |
| *TMEM213* | Collecting duct intercalated cells |

References of each gene were shown in manuscript.
